# Human rights among Korean dental hygiene students in hospital clinical practice

**DOI:** 10.1186/s12909-024-05655-0

**Published:** 2024-06-17

**Authors:** Yoon-Ah Won, Hie-Jin Noh

**Affiliations:** Department of Dental Hygiene at the College of Software and Digital Healthcare Convergence, Yonsei University, Wonju Gangwon-Do, Korea

**Keywords:** Dental institution, Dental hygiene student, Human rights, Hospital clinical practice

## Abstract

**Background:**

The human rights of dental hygiene students should be guaranteed during practice at medical institutions for their mental and physical health as well as professionalism, for patient safety. Safe and well guaranteed clinicians can perform their work in a more stable way. This study investigated the human rights circumstances of dental hygiene students during their hospital clinical practice at dental institutions.

**Methods:**

This study used a cross-sectional survey design. Convenience sampling was conducted on 121 third- and fourth-year dental hygiene students from universities in Seoul, Gyeonggi, and Gangwon. The survey used the Human Rights Indicators for Dental Hygiene Students to investigate the rights to safety, equality, and personality to understand participants’ experiences of guaranteed fundamental rights. Data were collected from October 31 to November 8, 2019. A chi-square test was used to assess differences in experience according to general characteristics.

**Results:**

During dental hygiene practice at dental institutions, less than 50% of students felt safe. When human rights violations occurred in dental institutions, only 42.4% of students received guidance on response measures from their universities. While 72.1% of students who practiced at dental university hospitals were given information on first aid supplies (facilities) within dental institutions, only approximately 45% of students who practiced at lower-scale dental institutions were given this information (*p* < 0.05). Regarding equality rights, only 52.5% of trainees reported that they had received equal treatment from healthcare workers during hospital clinical practice.

**Conclusions:**

During dental practice at dental institutions, Korean dental hygiene students confirmed that human rights (including safety rights, equality rights, and personality rights) were guaranteed to varying degrees. Dental hygiene students’ rights during hospital clinical practice in dental institutions should be guaranteed across institutions regardless of their scale. This is necessary for dental hygiene students’ human rights and safe policies and guidelines in dental institution clinical practice and regular monitoring systems.

## Background

### Hospital clinical practice in Korea

Hospital clinical practice in dental institutions forms part of the dental hygiene curricula in South Korea and is implemented during the mid-semester, or in the summer and winter vacations [1]. Students observe treatment procedures in various departments, such as prosthodontics, endodontics, and orthodontics in dental clinics, dental hospitals, and university dental hospitals, or participate in practice-based learning as assistants [2]. Hospital clinical practice in dental institutions in Korea comprises 8–17 weeks [1]. During hospital clinical practice, students meet various visitors and healthcare workers and take on the additional role of assistants [3–6].

### Human rights of students

Trainee students have the following guaranteed rights, among others, according to the Student Human Rights Ordinance: the right not to be discriminated against, protection from violence, safety rights, the right to learn, the right to be educated in a pleasant environment, and the right to freely express opinions [7, 8]. Human rights are the natural rights human beings have, and include the rights to safety, equality, and respect [9, 10]. When these rights are restricted, human rights violations occur.

**Fig. 1:**
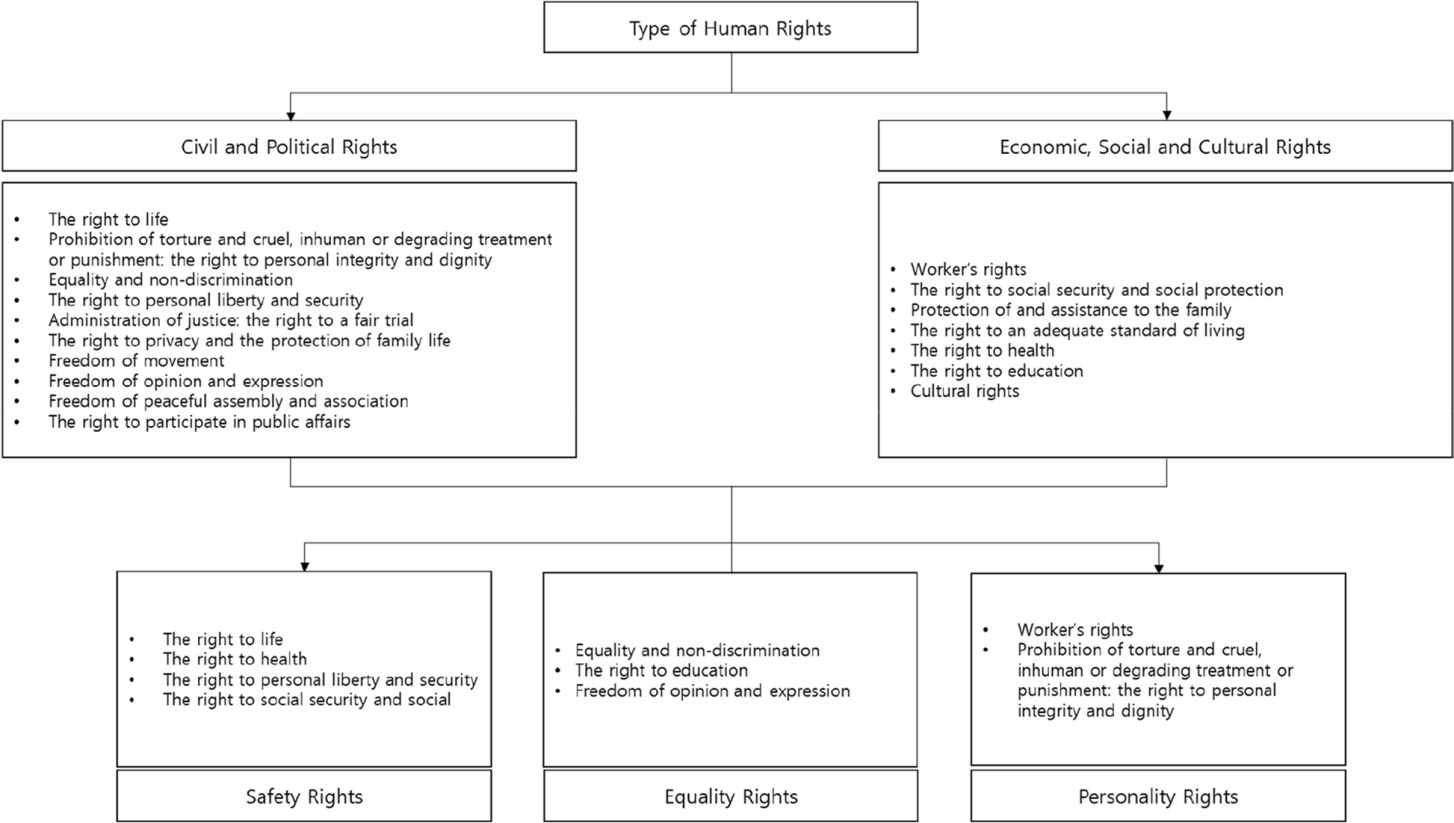
Conceptual framework for guaranteeing basic rights (Won et al. [11], adapted with permission)

Won et al. [11] classified human rights into three categories focusing on content related to the practices of dental hygiene students in dental institutions, according to the types of human rights presented in the Universal Declaration of Human Rights [9] and the International Covenant on Civil and Political Rights and Economic, Social and Cultural Rights [10] (see Fig. 1):

1. Safety rights: The right to be protected in life, liberty, and property, and to be protected from various dangers [9, 10]. In this study, safety rights are the right to be safe from external risks, such as natural disasters, and from risks to one’s life and body caused by others during hospital clinical practice in dental institutions.
2. Equality rights: The right to be treated equally in terms of race, sex, and religion [9, 10]. In this study, it refers to the right to enjoy equality without any kind of distinction based on sex, language, race, and so on, during hospital clinical practice at dental institutions.
3. Personality rights: The right to be respected and recognized by others [9, 10]. In this study, it refers to the right to be protected from various types of violence during practice at dental institutions.

### The human rights situation in hospital clinical practice

The bullying, violence, and infringement of healthcare workers within medical institutions are ongoing problems and can cause serious safety and mental and physical health risks, as well as impact the organization and society as a whole [12, 13]. Some healthcare students are alienated from clinical settings during their hospital clinical practice and do not receive guidelines or guidance for dealing with harassment or discrimination within their institutions [4–6, 14]. Students may also be likely to experience human rights violations owing to a lack of experience, unfamiliarity with the clinical environment, and perceived lack of power in themselves [15]. Tee et al. [16] reported that approximately 77% of nursing students at medical institutions had negative experiences concerning equality and personality rights, such as experiencing physical violence, and 33.0% were denied learning opportunities. Sinclair et al. [17] reported that 86% of nursing students experienced an unsafe practice environment. Negative experiences during hospital clinical practice in medical institutions cause stress [18]; induce depression, helplessness, and anxiety; and negatively impact self-esteem, career identity, and personal task performance, such as disengagement with practice or giving up the major [16]. Identifying strategies to prevent and resolve human rights violations to retain future dental hygienists is necessary [19].

The human rights situation of students engaged in hospital clinical practice must be investigated, identified, and monitored to create a healthy environment for hospital clinical practice [20]. Some studies have examined the human rights situation of students engaged in hospital clinical practice at medical institutions, such as medical or nursing colleges in Korea [6, 21]. However, it is still uncommon for institutions to develop guidelines to guarantee students’ human rights, including those of dental hygiene students, in hospital clinical practice environments. Therefore, to determine dental hygiene students’ human rights situation during hospital clinical practice in dental institutions, this study examined students’ experiences with guaranteed fundamental rights.

## Methods

### Study design

This study employed a cross-sectional survey design to determine the human rights situation of dental hygiene students during hospital clinical practice at dental institutions. The study was conducted between June 30, 2019 and December 16, 2019.

### Sampling procedures

The respondents were recruited from three- and four-year colleges with dental hygiene departments, located in Seoul, Gyeonggi, and Gangwon; participation was voluntary. The codes for universities located in Gangwon, Seoul, and Gyeonggi were A, B, and C, respectively. Using G*power 3.1.9.7 [22], the minimum sample size necessary for chi-square testing was calculated with an effect size = 0.3, significance level (α) = 0.05, power (1-β) = 0.8, and df = 2. Accordingly, 108 participants were required. This study used a convenience sample of senior students (third and fourth years) from dental hygiene departments; they had selected dental institutions in Seoul and Gyeonggi for their main hospital clinical practice. Considering a dropout rate of approximately 10%, 121 participants were finally selected for the analysis. The purpose and method of the study was explained to them, and their informed consent was obtained before conducting the survey.

### Data collection

This study used Won et al.’s Human Rights Indicators for Dental Hygiene Students [11] to determine dental hygiene students’ human rights situation during hospital clinical practice in dental institutions. This indicator comprises 11 questions and is reliable in terms of construct validity (Kaiser–Meyer–Olkin = 0.677) and internal consistency (Cronbach’s α = 0.734) [11]. The three items on fundamental rights were developed in the area of general human rights [9, 10], focusing on content related to dental hygiene students’ hospital clinical practice in dental institutions. In this study, 10 questions were selected and used according to the research purpose, and the total Cronbach’s α was 0.725. The questionnaire collected information on participants’ general characteristics and their experience of guaranteed fundamental rights.

The survey was conducted from October 31 to November 8, 2019. The students who agreed to participate were asked to complete a self-report questionnaire, which took approximately 5 min. These questionnaires were collected directly by the researchers. Completed questionnaires were retrieved from 121 participants, out of which 118 were used for the final analysis, as three participants’ questionnaires were excluded owing to insincere responses.

### Tools

#### General characteristics

Data on sex, duration of the most recent hospital clinical practice experience, dental training institution types, and training instructor type were collected to identify the participants’ general characteristics.

#### Safety rights

The items on safety rights included content related to the guaranteed rights of students during their preparation for hospital clinical practice and subsequent participation. A total of six items were rated on a dichotomous “yes” or “no” scale.

#### Equality rights

Items regarding equality rights aimed to determine whether education was indiscriminately provided to all the students. A total of two items were rated on a dichotomous “yes” or “no” scale.

#### Personality rights

Personality rights were measured in terms of experiences of physical and sexual violence. A total of 2 items were rated on a dichotomous “yes” or “no” scale.

### Data analysis

The data were analyzed using SPSS 25.0 (IBM, USA), and descriptive statistics and frequency analysis were employed for each category and item. A chi-square test was conducted to determine the differences in participants’ experiences and general characteristics.

### Ethics issues

This study was approved by the Institutional Review Board of Wonju Severance Christian Hospital, Yonsei University (IRB No. CR319070). All methods were performed in accordance with the relevant guidelines and regulations (Declaration of Helsinki). The respondents were recruited directly from universities located in Seoul, Gyeonggi, and Gangwon, and participation was voluntary. The purpose and method of the study were explained to the participants, and informed consent was obtained before conducting the survey.

## Results

### Participants’ general characteristics

In total, 95.8% of the participants were female, and 25.4%, 28.8%, and 45.8% were from Schools A, B, and C, respectively. Regarding dental training institution types, 51.7% of participants received training at university dental hospitals, 21.2% at dental hospitals, 19.5% at general hospitals, and 7.6% at dental clinics. Most (99.2%) participants’ training instructors were dental hygienists. The results are summarized in Table 1.

**Table 1:** Participants’ general characteristics

Characteristics	Variable	n (%)
Sex	Male	5 (4.2%)
	Female	113 (95.8%)
School	A	30 (25.4%)
	B	34 (28.8%)
	C	54 (45.8%)
Duration of hospital clinical practice (weeks)	≤ 4	62 (52.5%)
	≥ 5	56 (47.5%)
Dental training institution types	Dental clinics	9 (7.6%)
	Dental hospitals	25 (21.2%)
	General hospitals	23 (19.5%)
	University dental hospitals	61 (51.7%)
Training instructor	Dental hygienist	117 (99.2%)
	Other	1 (0.8%)
Total		118 (100%)

### Participants’ experience of guaranteed fundamental rights

#### Safety rights

**Fig. 2:**
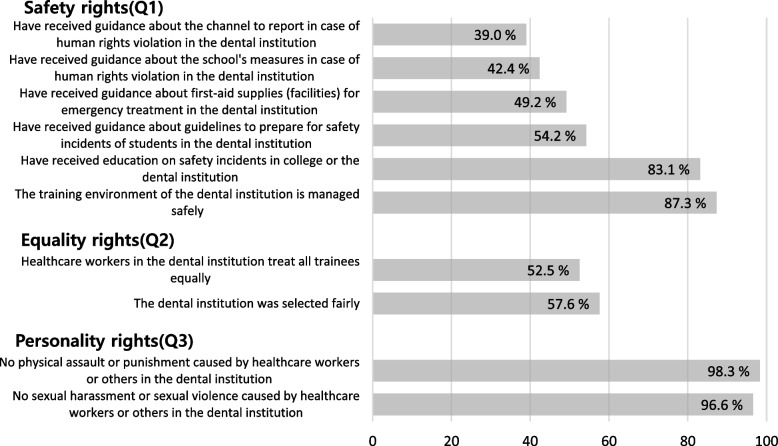
Dental hygiene students’ experience of guaranteed fundamental rights (*N* = 118)

Overall, 87.3% of the participants reported that their training institutions’ hospital clinical practice environment was managed safely. However, only 39.0% and 42.4% of the students received information on reporting channels and appropriate measures in cases of human rights violations, respectively, and 54.2% and 49.2% affirmed that they had received guidelines on preparing for safety incidents and guidance regarding first-aid supplies (facilities), respectively (see Fig. 2).

#### Equality rights

Overall, 52.5% of the participants reported that they had not been discriminated against during their hospital clinical practice in dental institutions (see Fig. 2).

### Personality rights

Overall, 96.6% of the participants responded that they had never experienced sexual harassment or sexual violence at the training institution, and 98.3% reported that they had not experienced physical assault or punishment (see Fig. 2).

### Differences in participants’ experiences regarding the guarantee of safety rights according to general characteristics

A significant difference was observed in the guidance on first-aid supplies (facilities) (Q1–3) within dental training institution types (*p* < 0.05). While 72.1% of trainee students at dental university hospitals received guidelines, only 44.4% received them at dental clinics, 39.1% at general hospitals, and 28.0% at dental hospitals (see Table 2). Furthermore, the following percentages of students confirmed having received guidelines to prepare for safety incidents (see Table 2): 93.4% at university dental hospitals, 87.0% at general hospitals, 84.0% at dental hospitals, and only 55.6% at dental clinics (*p* < 0.05).

**Table 2:** Differences in participants’ experiences with guarantee of safety rights according to general characteristics (*N* = 118)

Characteristic	Scope	n	Q1-1	*p*	Q1-2	*p*	Q1-3	*p*	Q1-4	*p*	Q1-5	*p*	Q1-6	*p*
School	A	30	24 (80.0%)	0.326	17 (56.7%)	0.067	19 (63.3%)	0.493	27 (90.0%)	0.076	18 (60.0%)	0.002^*^	12 (40.0%)	0.805
	B	34	31 (91.2%)		11 (32.4%)		185 (52.9%)		26 (76.5%)		6 (17.6%)		16 (47.1%)	
	C	54	43 (79.6%)		30 (55.6%)		27 (50.0%)		50 (92.6%)		22 (40.7%)		22 (40.7%)	
Dental training institution type	Dental clinics	9	5 (55.6%)	0.139	3 (33.3%)	0.287	4 (44.4%)	0.001^*^	5 (55.6%)	0.015^*^	3 (33.3%)	0.039^*^	3 (33.3%)	0.447
	Dental hospitals	25	21 (84.0%)		9 (36.0%)		7 (28.0%)		21 (84.0%)		4 (16.0%)		8 (32.0%)	
	General hospitals	23	19 (82.6%)		12 (52.2%)		9 (39.1%)		20 (87.0%)		9 (39.1%)		9 (39.1%)	
	University dental hospitals	61	53 (86.9%)		34 (55.7%)		44 (72.1%)		57 (93.4%)		30 (49.2%)		30 (49.2%)	
Duration of hospital clinical practice	≤ 4	62	53 (85.5%)	0.459	28 (45.2%)	0.361	36 (58.1%)	0.380	51 (82.3%)	0.084	24 (38.7%)	0.949	28 (45.2%)	0.519
	5 ≤	56	45 (80.4%)		30 (53.6%)		28 (50.0%)		52 (92.9%)		22 (39.3%)		22 (39.3%)	
Training instructor	Dental hygienist	117	97 (82.9%)	0.650	58 (49.6%)	0.323	64 (54.7%)	0.274	102 (87.2%)	0.702	46 (39.3%)	0.422	50 (42.7%)	0.389
	Other	1	1 (100%)		0 (0.0%)		0 (0.0%)		1 (100%)		0 (0.0%)		0 (0.0%)	

Q1 denotes the safety rights question (see Fig. 2), *p < 0.05 according to the chi-square test

### Differences in participants’ experience of guaranteed equality rights according to general characteristics

A total of 66.7% of the participants from School A, 55.6% from School C, and 35.3% from School B reported that the workers at the dental institutions treated all students equally (Q2–1, *p* < 0.05). There was no statistically significant difference by the type of dental training institution, but only 59.0% of students at dental university hospitals, 52.2% of those at general hospitals, 36.0% of those at dental hospitals, and 55.6% of those at dental clinics felt that they were treated equally (see Table 3).

**Table 3:** Differences in participants’ experience of guaranteed equality rights according to general characteristics (*N* = 118)

Characteristic	Scope	n	Q2-1	*p*	Q2-2	*p*
School	A	30	20 (66.7%)	0.001*	24 (80.0%)	< 0.05*
	B	34	12 (35.3%)		8 (23.5%)	
	C	54	30 (55.6%)		36 (66.7%)	
Dental training institution type	Dental clinics	9	5 (55.6%)	0.449	5 (55.6%)	0.442
	Dental hospitals	25	9 (36.0%)		11 (44.0%)	
					15 (65.2%)	
	General hospitals	23	12 (52.2%)		37 (60.7%)	
	University dental hospitals	61	36 (59.0%)			
Duration of hospital clinical practice	≤ 4	62	32 (51.6%)	0.091	32 (51.6%)	0.164
	≥ 5	56	30 (53.6%)		36 (64.3%)	
Training instructor type	Dental hygienist	117	62 (53.0%)	0.185	68 (58.1%)	0.242
	Others	1	0 (0.0%)		0 (0.0%)	

Q2 denotes the equality rights question (see Fig. 2), *p < 0.05 according to chi-square test

### Differences in participants’ experience of guaranteed personality rights according to general characteristics

Most of the students who engaged in hospital clinical practice reported that they had experienced neither physical assault or punishment (Q3–1) nor sexual harassment or sexual violence (Q3–2) perpetrated by dental healthcare or other workers in their dental institutions. However, some students claimed that their personality rights were not guaranteed (see Table 4).

**Table 4:** Differences in participants’ experience of guaranteed personality rights according to general characteristics (*N* = 118)

Characteristic	Scope	n	Q3-1	*p*	Q3-2	*p*
School	A	30	29 (96.7%)	0.981	29 (96.7%)	0.583
	B	34	33 (97.1%)		34 (100%)	
	C	54	52 (96.3%)		53 (98.1%)	
Dental training institution type	Dental clinics	9	9 (100%)	0.639	9 (100%)	0.593
	Dental hospitals	25	25 (100%)		25 (100%)	
	General hospitals	23	22 (95.7%)		23 (100%)	
	University dental hospitals	61	58 (95.1%)		59 (96.7%)	
Duration of hospital clinical practice	≤ 4	62	60 (96.8%)	0.917	61 (98.4%)	0.942
	5 ≤	56	54 (96.4%)		55 (98.2%)	
Training instructor type	Dental hygienist	117	113 (96.6%)	0.851	115 (98.3%)	0.895
	Other	1	1 (100%)		1 (100%)	

Q3 denotes the personality rights question (see Fig. 2), *p < 0.05 according to chi-square test

## Discussion

This study aimed to confirm the experience of dental hygiene students in guaranteeing their basic rights during hospital clinical practice at dental institutions. Although some areas of safety, equality, and personality rights were relatively well guaranteed, some students responded negatively.

Overall, 42.2% of the participants reported that they had received guidance on how to respond in the event of human rights violations. Warshawski [23] reported that 37.7% of nursing students and 7.3% of medical students received guidance on coping measures. Tee et al. [16] noted that students did not know where or how to report human rights violations. Additionally, they were unable to report violations for fear that no action would be taken or that they would be harmed, and they felt that reporting would not resolve the problem properly [16]. Some researchers have suggested that it is necessary to teach students to identify human rights violations and actively report them [24–26]. A medical office environment is likely to be exposed to various risks, such as sharp instruments, blood, and aerosols, which highlights the need for safety awareness and management [27–29]. However, in this study, only appsroximately 49% of all participants the students by size of each dental institution received safety accident education and felt that the practice environment was safely managed (see Table 2). Yoo and Oh [30] reported that those who received education had fewer safety incidents.

Compared with other trainee students, only 52.5% of the participants claimed that they had received equal treatment from healthcare workers during their hospital clinical practice in dental institutions (see Fig. 2). Son et al. [5] reported that some dental hygiene students received more discriminatory treatment than students from other schools who attended hospital clinical practice. Karatas et al. [31] reported that 78.1% of nursing students in hospital clinical practice reported experiencing discrimination perpetrated by healthcare workers and fellow students. As hospital clinical practice promotes positive learning through interactions between students and instructors, an important condition for successful hospital clinical practice is instructors treating students fairly [32, 33].

Regarding the guarantee of personality rights, over 96% of the students had not experienced physical or sexual violence (see Fig. 2), but personality rights were not guaranteed for approximately 2.5% of the students, highlighting a serious problem. Cho and Lee [34] reported that 11.5% of dental hygiene students experienced sexual harassment during hospital clinical practice in dental institutions. Lee et al. [35] reported that 21.1% of nursing students in hospital clinical practice experienced physical assault and 47.3% experienced sexual violence. Physical and sexual violence causes widespread psychological trauma, including anxiety and depression [36, 37]. It also leads to job dissatisfaction and decreased work quality and efficiency [36, 37], which can affect medical services. While only a few participants in this study experienced physical or sexual violence, additional research with more students is required to clarify the findings.

The seriousness of human rights violations within medical institutions, such as discrimination, bullying, violence, and safety, has been highlighted, and various studies have been conducted to resolve the challenges. Methods to resolve and prevent conflicts related to human rights violations include workshops and educational campaigns on team building, which can positively affect the environment [25, 26, 36–38]. Examples include a warning event policy that ensures early reporting without fear of threats, continuous monitoring and practice-based training and coaching, educational campaigns to increase awareness of human rights issues, and workshops to understand the other party through interactive role-play and games [25, 26, 36–38]. Researchers have reported that these methods can increase awareness of human rights issues, change the attitudes of perpetrators, and encourage different ways of interacting [25, 26, 36–38]. According to the results of this study, most rights related to safety, equality, and personality were relatively well guaranteed, but some students felt that they were not, and their views should not be taken lightly. As efforts are being made in various fields to improve the human rights issue of trainee students, we recommend ongoing review and monitoring of the dental hygiene community as well as further research on policy development.

This study identified the human rights condition of dental hygiene students during hospital clinical practice in dental institutions by identifying and applying certain human rights indicators. However, as participants were recruited using convenience sampling from specific regions, generalizing the results to all dental hygiene students in Korea should be undertaken with caution. Moreover, participants’ responses to the questionnaire might have been insufficient or insincere owing to the sensitive nature of certain questions. As colleges select different dental institutions as hospital clinical practice sites, the differences in characteristics among institutions might also have affected the results of this study. Further efforts should be made to examine the condition of human rights of dental hygiene students across Korea during hospital clinical practice and improve their situation.

## Conclusions

The human rights of dental hygiene students at dental institutions were found to be relatively well guaranteed, but some results were negative and varied. Regardless of the size of the dental institution, the problems were similar to those observed in other healthcare fields. To resolve the challenges, additional research is needed for regular monitoring through human rights surveys, reviewing the corresponding approaches, and establishing policies and systems to ensure the basic rights of dental hygiene students.

## Data Availability

The dataset used and analyzed during the current study is available from the corresponding author on reasonable request.
